# Distinct effects of IPSU and suvorexant on mouse sleep architecture

**DOI:** 10.3389/fnins.2013.00235

**Published:** 2013-12-10

**Authors:** Daniel Hoyer, Thomas Dürst, Markus Fendt, Laura H. Jacobson, Claudia Betschart, Samuel Hintermann, Dirk Behnke, Simona Cotesta, Grit Laue, Silvio Ofner, Eric Legangneux, Christine E. Gee

**Affiliations:** ^1^Neuroscience, Novartis Institutes for BioMedical ResearchBasel, Switzerland; ^2^Department of Pharmacology and Therapeutics, Faculty of Medicine, Dentistry and Health Sciences, The University of MelbourneParkville, VIC, Australia; ^3^Center of Behavioral Brain Sciences, Institute for Pharmacology and Toxicology, Otto-von-Guericke University MagdeburgMagdeburg, Germany; ^4^The Florey Institute of Neuroscience and Mental Health, The University of MelbourneParkville, VIC, Australia; ^5^Global Discovery Chemistry, Novartis Institutes for BioMedical ResearchBasel, Switzerland; ^6^Metabolism and Pharmacokinetics, Novartis Institutes for BioMedical ResearchBasel, Switzerland; ^7^Translational Medicine, Novartis Institutes for BioMedical ResearchBasel, Switzerland; ^8^Center for Molecular Neuroscience Hamburg, Institute for Synaptic PhysiologyHamburg, Germany

**Keywords:** orexin receptor antagonist, insomnia, pharmacology, REM and NREM sleep

## Abstract

Dual orexin receptor (OXR) antagonists (DORAs) such as almorexant, SB-649868, suvorexant (MK-4305), and filorexant (MK-6096), have shown promise for the treatment of insomnias and sleep disorders. Whether antagonism of both OX_1_R and OX_2_R is necessary for sleep induction has been a matter of some debate. Experiments using knockout mice suggest that it may be sufficient to antagonize only OX_2_R. The recent identification of an orally bioavailable, brain penetrant OX_2_R preferring antagonist 2-((1*H*-Indol-3-yl)methyl)-9-(4-methoxypyrimidin-2-yl)-2,9-diazaspiro[5.5]undecan-1-one (IPSU) has allowed us to test whether selective antagonism of OX_2_R may also be a viable strategy for induction of sleep. We previously demonstrated that IPSU and suvorexant increase sleep when dosed during the mouse active phase (lights off); IPSU inducing sleep primarily by increasing NREM sleep, suvorexant primarily by increasing REM sleep. Here, our goal was to determine whether suvorexant and IPSU affect sleep architecture independently of overall sleep induction. We therefore tested suvorexant (25 mg/kg) and IPSU (50 mg/kg) in mice during the inactive phase (lights on) when sleep is naturally more prevalent and when orexin levels are normally low. Whereas IPSU was devoid of effects on the time spent in NREM or REM, suvorexant substantially disturbed the sleep architecture by selectively increasing REM during the first 4 h after dosing. At the doses tested, suvorexant significantly decreased wake only during the first hour and IPSU did not affect wake time. These data suggest that OX_2_R preferring antagonists may have a reduced tendency for perturbing NREM/REM architecture in comparison with DORAs. Whether this effect will prove to be a general feature of OX_2_R antagonists vs. DORAs remains to be seen.

## Introduction

Since the link between the hypocretin/orexin system and sleep disorders was discovered (Chemelli et al., 1999; Lin et al., 1999; Nishino et al., 2000), there has been much interest in developing orexin receptor antagonists (ORAs) for the treatment of insomnia. Several dual orexin receptor antagonists (DORAs) have now been tested in the clinic and have demonstrated sleep inducing properties in healthy volunteers and/or in patients suffering from insomnia (Brisbare-Roch et al., 2007; Winrow et al., 2011; Bettica et al., 2012a). Whereas ORAs are expected to be without the side effects characteristic of currently available treatments, questions about mechanism related safety have accompanied their development. As the lack of orexin signaling causes narcolepsy with cataplexy, there is concern that DORAs may induce sudden loss of motor control or sleep attacks. Whereas the therapeutic potential for treating insomnia with ORAs is undisputedly high, there is less consensus regarding the necessity of targeting both receptors and whether selective antagonists might reduce potential safety concerns without loss of efficacy (Mieda et al., 2013).

Several lines of evidence suggest that selective OX_2_R antagonists may be sufficient for sleep induction and may have a reduced tendency for induction of cataplexy and/or narcolepsy in comparison with DORAs. In knockout mice, the sleep inducing properties of the DORA almorexant require intact OX_2_Rs but not OX_1_Rs (Mang et al., 2012). Also, selective OX_2_R antagonists induce sleep in rats and mice, whereas OX_1_R selective antagonists do not [(Dugovic et al., 2009; Steiner et al., 2013); but see Morairty et al. (2012)]. Together these findings strongly suggest that antagonizing OX_2_R may be sufficient for sleep induction.

In humans, orexin deficiency leads to narcolepsy with cataplexy (Nishino et al., 2000). The narcolepsy/cataplexy phenotype is mimicked in mice lacking orexin peptides, lacking orexinergic neurons or mice lacking both orexin receptors (OXR) (Chemelli et al., 1999; Hara et al., 2001; Kalogiannis et al., 2011). Although mice lacking OX_2_R also have sleep attacks, the incidence of cataplexy in these mice is close to null (Willie et al., 2003). Thus, there may be a reduced risk of inducing cataplectic events when only OX_2_R are antagonized. In dogs, mutations in OX_2_R alone are sufficient to cause narcolepsy with cataplexy. Interestingly, sporadic narcolepsy with cataplexy in dogs is associated with orexin deficiency resulting in more severe symptoms than the OX_2_R mutations (Baker et al., 1982; Ripley et al., 2001). It is tempting to speculate that OX_2_R antagonists might be less prone to induce symptoms similar to either narcolepsy or cataplexy than DORAs even in this very sensitive species. To date, although functionally relevant polymorphisms of the OXRs have been described in humans (Thompson et al., 2004; Rainero et al., 2008; Annerbrink et al., 2011), mutations of the receptors have not been linked to narcolepsy/cataplexy. Rather loss of the orexinergic neurons or, extremely rarely, mutations resulting in loss of the peptides have been reported to be the underlying cause (Peyron et al., 2000; Thannickal et al., 2000). Together, such findings suggest it may be a general rule that loss of OX_2_R signaling has a reduced propensity for inducing narcolepsy and/or cataplexy as compared to loss of the entire orexin signaling pathway.

We have therefore been interested in determining whether antagonists of OX_2_R are comparable to DORAs in their ability to induce sleep. Recently, we identified the orally bioavailable, brain penetrating OX_2_R preferring antagonist IPSU that induces sleep when administered at the start of the active (dark) phase in mice (Betschart et al., 2013). The aim of the present study was to investigate whether IPSU and the DORA suvorexant affect the natural sleep architecture, by testing low doses of the two compounds during the light phase when mice are primarily inactive and spend a high proportion of the time sleeping.

## Methods

All experiments were performed according to Swiss guidelines and law and were approved by the Veterinary Authority of Basel-Stadt, Switzerland. Every effort was made to minimize the number of animals used and to minimize any pain or discomfort. Male C57Bl/6 mice weighing 25–35 g were single or group-housed on wood shavings in type II (14 × 16 × 22 cm) and type III (15 × 22 × 37 cm) cages, respectively. Each cage contained a nest box, a piece of wood and tissue paper nesting materials, and animals had access to food and water *ad libitum*. The housing cages were placed in a temperature and humidity controlled room (20–24°C, 45% humidity) with a light/dark cycle of 12:12 (lights on at 03:00, max 80 Lux).

Suvorexant and IPSU were both synthesized and purified *in house* according to published procedures (Cox et al., 2008; Betschart et al., 2013). We selected doses that were effective at promoting sleep in mice for the first 4 h when administered at the start of the dark phase [Betschart et al. (2013) and unpublished observations]. At the mouse OXRs, IPSU has about 6.2× higher affinity at OX_2_R than OX_1_R (pKd OX_1_R 6.34, OX_2_R 7.23) whereas suvorexant is about 6.5× more potent at OX_1_R than OX_2_R [pKd OX_1_R 8.77, OX_2_R 8.06; FLIPR assay Callander et al. (2013)]. Both compounds are highly brain penetrant. One hour after oral dosing of 50 mg/kg, brain levels reached 8778 pmol/g for IPSU and 10329 pmol/g for suvorexant giving free levels of 53.6 and 67.0 pmol/g, respectively (Betschart et al., 2013). In the present study we decided to dose IPSU at the previously effective dose of 50 mg/kg and to reduce suvorexant to 25 mg/kg to better match the estimated OX_2_R occupancy. At 1 h following 25 mg/kg suvorexant brain levels reached 3605 pmol/g and free levels were therefore 23.4 pmol/g. Estimating the available antagonist concentrations to be 53.6 and 23.4 nM for IPSU and suvorexant, we estimated receptor occupancies according to:

Bound =Bmax/(1+Kd/L)

where Bmax is 100%, Kd the affinity from the FLIPR assay, and L the free brain concentrations at the doses tested.

The expected occupancies of suvorexant are therefore 93% at OX_1_R, 73% at OX_2_R and for IPSU 11% at OX_1_R and 48% at OX_2_R. These values are similar to the effective values for sleep induction at OX_2_R reported by Gotter et al. (2013).

### Implantation of electrocorticogram/electroencephalogram (EEG) and electromyogram (EMG) electrodes

Mice were administered buprenorphine (0.05 mg/kg s.c.) 1 h before surgery, anesthetized with ketamine/xylazine (110 mg/kg, 10:1, i.p.) and placed in a stereotaxic frame. The skull was exposed and four miniature stainless steel screws (SS-5/TA Science Products GmbH, Hofheim, Germany) attached to 36-gauge, Teflon-coated solid silver wires were placed in contact with the frontal and parietal cortex (3 mm posterior to bregma, ±2 mm from the sagittal suture) through bore holes. The frontal electrodes served as reference. The wires were crimped to a small 6-channel connector (CRISTEK Micro Strip Connector) that was affixed to the skull with dental acrylic. Electromyograph (EMG) signals were acquired by a pair of multistranded stainless steel wires (7SS-1T, Science Products GmbH, Hofheim, Germany) inserted into the neck muscles and also crimped to the headmount. After surgery, mice were kept singly in cages and allowed to recover on a heating pad. Buprenorphine, 0.05 mg/kg, s.c. was given 8 and 16 h after surgery to control pain. After 24 h, the mice were housed with their former cage mates and allowed to recover for 2 weeks.

### Sleep studies

Mice were habituated to individual cages in a sound-attenuated recording chamber for 6–10 days (lights on 03:00, lights off 15:00, max 80 lux) at a temperature of about 23°C. During the studies, mice had access to food and water *ad libitum*, to one sheet of nesting paper and a piece of wood but no nesting box. Mice were weighed and attached to recording cables that connected their headmounts to a commutator (G-4-E, Gaueschi) allowing free movement in the experiment boxes, 1 day before beginning the experiment. The recording chamber was opened each day during the light period between 08:00 and 09:00 to care for the mice and all experimental manipulations and oral applications were performed in a time window of 5–15 min before the start of the recordings at 09:00, exactly 6 h after lights on. Day 1, the mice were manipulated and habituated to the oral application syringe. Day 2, they received vehicle (methylcellulose 0.5%, 10 mL/kg, per os). Day 3, 50 mg/kg IPSU or 25 mg/kg suvorexant was administered *per os*. Recordings began at 09:00 (hour 0) and continued for 23 h. The experimental chamber was secured about 5 min prior to start of the recordings and the mice remained undisturbed for the next 23 h. On Day 4, the mice were returned to their normal housing cages for at least 2 weeks before returning to the experiment.

EEG/EMG signals were amplified using a Grass Model 78D amplifier (Grass Instrument CO., Quincy, MA, USA), analog filtered (EEG: 0.3–30 Hz, EMG: 5–30 Hz) and acquired using Harmonie V5.2 (acquisition frequency: 200 Hz with calibration the first day, record duration: 23h). Animals were video recorded during data collection, using an infrared video camera and locomotor activity was detected using infrared sensors (InfraMot Infrared Activity Sensor 30-2015 SENS, TSE Systems) placed in the roof of the boxes. Activity signals were acquired in 10 s intervals by the software Labmaster V2.4.4. EEG/EMG and activity recordings were imported into and scored in 10 s epochs using the rodent scoring module of Somnologica into wake, NREM sleep and REM sleep. Epochs during which there were state transitions were scored as the state present for at least 50% of the epoch. The time in each state per hour was calculated and mean ± s.e.m. is shown. Restricted maximum likelihood analysis (REML) was applied to the data from the first 6 h to determine if there was a statistically significant effect of treatment or a significant interaction between treatment and hour. When either treatment or the interaction was significant (*p* < 0.05), *post-hoc* Fisher's Least Significant Difference (LSD) tests were applied to determine hour by hour where there were significant differences between the vehicle and compound days.

The first 2 h following drug treatment were, in addition, manually scored to assess sleep-wake transitions including very short awakenings i.e., those with durations of 1–5 s that are often seen in mice and which would not be detected using 10 s epochs.

## Results

**Figure 1:**
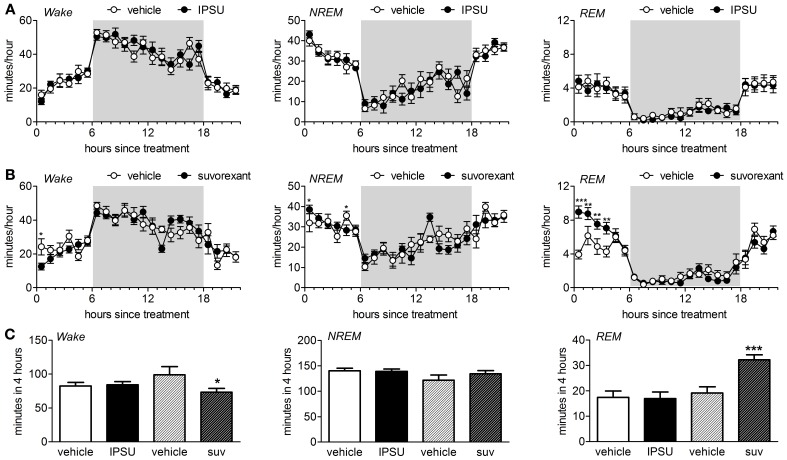
**Sleep architecture during the inactive period is perturbed by a DORA but not by an OX_2_R antagonist in C57Bl/6 mice. (A)** EEG/EMG/motility signals were used to score vigilance states into wake, NREM and REM beginning from time 0, 6 h into the light period. Vehicle (0.5% methylcellulose in water) or 50 mg/kg IPSU were applied *per os* 5–15 min prior to start of the recordings on successive days (*n* = 11). The mean ± s.e.m. minutes per hour spent in each stage are shown. Shading indicates the dark period. **(B)** Vehicle (0.5% methylcellulose in water) or 25 mg/kg suvorexant were applied *per os* 5–15 min prior to start of the recordings on successive days (*n* = 11). ^*^p < 0.05, ^**^p < 0.01, ^***^p < 0.001 Fisher's LSD. **(C)** Quantification of the effect of IPSU and suvorexant on wake, NREM and REM during the first 4 h post-treatment. ^*^p < 0.05, ^***^p < 0.001 paired *t-test* drug vs. vehicle.

The ORA IPSU (50 mg/kg) had no effect on the sleep architecture of mice when administered during the middle of the light phase (Figures 1A,C). The amount of time spent in wake, NREM and REM were unchanged following administration of IPSU relative to the previous day when vehicle was administered [treatment: wake *F*_(1, 120)_ = 0.002, *p* = 0.96, NREM *F*_(1, 120)_ = 0.002, *p* = 0.97, REM *F*_(1, 120)_ = 0.001, *p* = 0.97, treatment × hour: wake *F*_(5, 120)_ = 0.59, *p* = 0.71, NREM *F*_(5, 120)_ = 0.56, *p* = 0.73, REM *F*_(5, 120)_ = 0.44, *p* = 0.82, REML].

In contrast, the DORA suvorexant (25 mg/kg) slightly decreased time spent in wake, had weak effects on time in NREM but strongly increased the amount of time spent in REM sleep during the first 4 h immediately following application [Figures 1B,C, treatment: wake *F*_(1, 120)_ = 3.4, *p* = 0.066, NREM *F*_(1, 120)_ = 0.44, *p* = 0.51, REM *F*_(1, 120)_ = 28.9, *p* < 0.001, treatment × hour: wake *F*_(5, 120)_ = 2.31, *p* = 0.048, NREM *F*_(5, 120)_ = 1.76, *p* = 0.13, REM *F*_(5, 120)_ = 3.37, *p* = 0.007, REML].

Manual rescoring of the first 2 h after vehicle or drug application confirmed that the automatic scoring was in excellent agreement when amount of time in each stage was compared, better than 90% as previously reported by us and others (Pick et al., 2011; Mang et al., 2012). The number of awakenings was, however, different. Very short awakenings were found within epochs scored as NREM (Léna et al., 2004). We therefore used the results from the manual scoring to quantify the latency to NREM or REM and the sleep-wake transitions (Table 1).

**Table 1:** **Effect of the OX_2_R preferring antagonist IPSU and the DORA, suvorexant on latency to sleep and sleep to wake transitions during the first 2 h after administration during the inactive period**.

	**Vehicle**	**IPSU**	**Diff**	**Vehicle**	**Suvorexant**	**Diff**
Latency to NREM (minutes)	9.5 ± 3.0	2.6 ± 1.0	−7.0 ± 3.3	13.1 ± 5.9	4.8 ± 1.9	−8.3 ± 6.3
Latency to REM (minutes)	22.1 ± 3.4	14.5 ± 2.3	−7.6 ± 4.5	37.0 ± 10.1	8.4 ± 2.3^*^	−28.6 ± 8.8
Awakenings from NREM	25.1 ± 2.6	25.3 ± 2.0	0.2 ± 2.4	19.4 ± 1.6	18.6 ± 1.9	−0.8 ± 2.4
Awakenings from REM	10.2 ± 0.9	10.8 ± 1.1	0.6 ± 1.1	8.2 ± 1.4	15.6 ± 1.2^**^	7.4 ± 1.6
Short awakenings from NREM (<5 s)	23.55 ± 2.8	26.5 ± 4.3	2.9 ± 1.9	25.7 ± 4.0	26.6 ± 3.1	0.9 ± 2.1
Total awakenings	58.8 ± 4.7	62.6 ± 5.1	3.7 ± 2.7	53.3 ± 4.9	60.8 ± 3.8^***^	7.5 ± 1.9

* *p < 0.05*,

** *p < 0.01*,

*** *p < 0.001, paired t-test*.

Both IPSU and suvorexant showed a tendency to shorten the latency to NREM and REM sleep but only the shortening of latency to REM by suvorexant was significantly different from the vehicle. Awakenings from NREM were unaffected by either compound. Whereas awakenings from REM were significantly increased by suvorexant, the OX_2_R antagonist IPSU had no effect.

## Discussion

Whereas IPSU did not perturb the normal sleep pattern of mice during the inactive period, suvorexant profoundly altered the sleep pattern, doubling the time spent in REM. The latency to REM and number of awakenings from REM was also selectively increased by suvorexant but not by IPSU. This pattern is similar to that found when these compounds were dosed at the start of the dark (active) period in mice (Betschart et al., 2013). Suvorexant promoted sleep primarily by increasing REM with small effects on NREM and IPSU promoted sleep primarily by promoting NREM and to a lesser degree REM (Betschart et al., 2013). The stronger enhancement of REM vs. NREM by suvorexant has also been seen in rats and in both healthy humans and humans suffering from insomnia (Winrow et al., 2011; Herring et al., 2012; Sun et al., 2013). Interestingly, our findings indicate that this DORA influences REM independently of the circadian dosing time, increasing the time in REM during both the light (active) phase and dark (active) phase whereas, at the dose tested, the OX_2_R preferring antagonist IPSU influences sleep only during the active phase in mice.

Influencing the balance between REM and NREM is an area of potential differentiation between OX_2_R preferring antagonists and DORAs. Classic benzodiazepines, “Z drugs” such as zolpidem, and antidepressants are well known for suppressing REM sleep whereas ORAs certainly lack this property. Although IPSU is not highly selective for OX_2_R vs. OX_1_R at mouse receptors (~6.2×), the opposite is true for suvorexant, which prefers mouse OX_1_R vs. OX_2_R (~6.5×, Betschart et al., 2013; Callander et al., 2013). Thus, the balance between antagonism of OX_1_R and OX_2_R may contribute to the differential effects of ORAs on sleep architecture. Our findings suggest that reducing the level of OX_1_R antagonism shifts the sleep balance toward NREM. Supporting this hypothesis, almorexant induces a greater REM increase in OX_1_R^−/−^ than in wildtype mice (Mang et al., 2012). Likewise, whereas both almorexant and the OX_2_R antagonist JNJ-10397049 increased NREM during the light phase in rats, only almorexant also increased REM, and co-application of an OX_1_R antagonist significantly reduced the NREM induced by the OX_2_R antagonist (Dugovic et al., 2009). For the most part, DORAs increase REM more than NREM in rodent studies when % increase is considered (Brisbare-Roch et al., 2007; Winrow et al., 2011; Betschart et al., 2013; Black et al., 2013). However, the contribution of REM as a proportion of total sleep time varies for different compounds. For example, in mice almorexant-induced increases in REM remain within the proportion seen during normal sleep, even at high doses (Mang et al., 2012), whereas suvorexant increases REM proportion much above that seen during normal sleep (Betschart et al., 2013). Almorexant is unusual among the DORAs in that it appears to become a somewhat OX_2_R preferring antagonist *in vivo*. The *ex vivo* occupancy of almorexant was found to be about 2x higher and much longer lasting at OX_2_Rs (>12 h) vs. OX_1_Rs (Morairty et al., 2012). This preference is most likely driven by the unusual kinetics (Malherbe et al., 2009; Mang et al., 2012; Callander et al., 2013) so that with short exposures the compound may act as a DORA, and when equilibrium is allowed to be reached almorexant has functional selectivity for OX_2_Rs. Evidence against our hypothesis includes the description of a newer DORA that increases NREM preferentially in rats (Sifferlen et al., 2013). The structure of this compound is quite similar to that of almorexant but whether the kinetics also bias it toward OX_2_R selectivity when equilibrated is unknown. Interestingly, species differences also exist in the effects of DORAs on sleep architecture. In dogs, suvorexant predominately increased NREM (Winrow et al., 2011). The effects of DORAs on REM in humans appear to more closely mimic the effects in rodents rather than dogs as in the clinical settings, REM was preferentially enhanced with SB-649868 (Bettica et al., 2012a,b,c) and suvorexant (Herring et al., 2012; Sun et al., 2013). With the limited data available, whether the differential effects of the DORAs and OX2R antagonists on sleep architecture are in fact due to differences in receptor affinity/occupancy or are due to other factors such as compound class and species, remains to be seen as more compounds from different chemical classes are developed.

Why might a DORA be expected to influence REM more strongly than an OX_2_R preferring antagonist? Intracerebroventicular or local application of orexin-A in the highly OX_1_R expressing locus coeruleus reduces REM sleep, an effect that is blocked by the OX_1_R antagonist SB-334867 (Smith et al., 2003; Mieda et al., 2011). Additionally, knock-down of OX_1_R receptors in the locus coeruleus selectively increases REM, without affecting NREM during the active phase (Chen et al., 2010). Interestingly, we did not see a similar dependence of circadian time on the REM enhancement by suvorexant. REM sleep is not however, exclusively modulated by OX_1_Rs. OX_2_R knockdown in the lateral pontomesencephalic tegmentum increased REM both during the active and inactive phases (Chen et al., 2013) and while OX_1_R antagonists alone generally do not induce REM (Steiner et al., 2013), they may increase REM on top of the effects of OX_2_R antagonists (Dugovic et al., 2009). Overall, both OX_1_R and OX_2_R when activated or down regulated in the appropriate regions appear to be able to modulate REM sleep. However, the modulatory role of OX_1_R on REM may be greater than that of OX_2_R.

In conclusion, we hypothesize that selective OX_2_R antagonists have potential for the treatment of insomnia and may prove to perturb sleep architecture to a lesser degree than some of the DORAs. More highly selective antagonists from different chemical classes will be required to test this hypothesis further.

## Author contributions

All authors read and commented on the manuscript. Daniel Hoyer and Claudia Betschart led the team and participated in writing the manuscript. Thomas Dürst planned and performed experiments and analyzed data. Claudia Betschart, Samuel Hintermann, Dirk Behnke, Silvio Ofner, and Simona Cotesta synthesized and/or designed compounds. Grit Laue performed *in vivo* analyses related to pharmacokinetics. Markus Fendt, Laura H. Jacobson, and Eric Legangneux contributed to experimental design and writing the manuscript. Christine E. Gee designed the study, performed analysis, and wrote the manuscript.

### Conflict of interest statement

All authors were or are employees of Novartis AG and may own stock in the company.
